# Comparison of Surgical Risk Scores in a European Cohort of Patients with Advanced Chronic Liver Disease

**DOI:** 10.3390/jcm12186100

**Published:** 2023-09-21

**Authors:** Lidia Canillas, Amalia Pelegrina, Elena Colominas-González, Aina Salis, César J. Enríquez-Rodríguez, Xavier Duran, Antonia Caro, Juan Álvarez, José A. Carrión

**Affiliations:** 1 Department of Medicine and Life Sciences, Universitat Pompeu Fabra, 08003 Barcelona, Spain; lcanillas@psmar.cat (L.C.); apelegrina@psmar.cat (A.P.); ecolominasgonzalez@psmar.cat (E.C.-G.); aina.salis01@estudiant.upf.edu (A.S.); cesarjesse.enriquez.rodriguez@psmar.cat (C.J.E.-R.); 2 Liver Section, Gastroenterology Department, Hospital del Mar, 08003 Barcelona, Spain; acaro@psmar.cat; 3 Hospital del Mar Medical Research Institute, 08003 Barcelona, Spain; jcalvarez@psmar.cat; 4 Department of General Surgery, Hospital del Mar, 08003 Barcelona, Spain; 5 Pharmacy Department, Hospital del Mar, 08003 Barcelona, Spain; 6 Department of Medicine, Universitat Autònoma de Barcelona, 08003 Barcelona, Spain; 7 Biostatistics Unit, Hospital del Mar Research Institute, 08003 Barcelona, Spain; xduran@researchmar.net; 8 Anesthesia Department, Hospital del Mar, 08003 Barcelona, Spain

**Keywords:** cirrhosis, advanced chronic liver disease, surgery, postoperative risk, mortality

## Abstract

Patients with advanced chronic liver disease (ACLD) or cirrhosis undergoing surgery have an increased risk of morbidity and mortality in contrast to the general population. This is a retrospective, observational study to evaluate the predictive capacity of surgical risk scores in European patients with ACLD. Cirrhosis was defined by the presence of thrombocytopenia with <150,000/uL and splenomegaly, and AST-to-Platelet Ratio Index >2, a nodular liver edge seen via ultrasound, transient elastography of >15 kPa, and/or signs of portal hypertension. We assessed variables related to 90-day mortality and the discrimination and calibration of current surgical scores (Child-Pugh, MELD-Na, MRS, NSQIP, and VOCAL-Penn). Only patients with ACLD and major surgeries included in VOCAL-Penn were considered (n = 512). The mortality rate at 90 days after surgery was 9.8%. Baseline disparities between the H. Mar and VOCAL-Penn cohorts were identified. Etiology, obesity, and platelet count were not associated with mortality. The VOCAL-Penn showed the best discrimination (C-statistic_90D_ = 0.876) and overall predictive capacity (Brier_90D_ = 0.054), but calibration was not excellent in our cohort. VOCAL-Penn was suboptimal in patients with diabetes (C-statistic_30D_ = 0.770), without signs of portal hypertension (C-statistic_30D_ = 0.555), or with abdominal wall (C-statistic_30D_ = 0.608) or urgent (C-statistic_180D_ = 0.692) surgeries. Our European cohort has shown a mortality rate after surgery similar to those described in American studies. However, some variables included in the VOCAL-Penn score were not associated with mortality, and VOCAL-Penn’s discriminative ability decreases in patients with diabetes, without signs of portal hypertension, and with abdominal wall or urgent surgeries. These results should be validated in larger multicenter and prospective studies.

## 1. Introduction

The increased life expectancy and aging of patients with chronic liver disease (CLD) entails an increased need for invasive procedures and surgeries in increasingly complex patients due to the addition of extrahepatic comorbidities. Patients with advanced chronic liver disease (ACLD) or cirrhosis undergoing surgery have an increased risk of morbidity and mortality in contrast to the general population [1].

Classically, the mortality risk after surgery has been related to liver function. High Child-Turcotte-Pugh (CTP) score values have consistently been associated with complications and mortality in surgical patients [2,3,4,5]. The Model for End-Stage Liver Disease (MELD) incorporates renal function and has been linearly correlated to postoperative mortality [6]. Through the years, there have been attempts to improve these predictions by incorporating variables related to comorbidity. However, the Charlson Comorbidity Index (CCI) [7] and the American Society of Anesthesiologists physical status classification system (ASA) [8] were designed for non-cirrhotic patients. In 2013, Jepsen et al. developed the Cirrhosis Comorbidity Score (CirCom) [9], a semi-quantitative scale that evaluates the long-term and non-surgical related mortality risk added to cirrhosis by other comorbidities. Nevertheless, prediction models designed to predict surgical risk are preferable. In the general population, one of the most used calculators is the National Surgery Quality Improvement Program (NSQIP) [10]. The NSQIP includes 20 variables, but only one (ascites in the previous 30 days) is related to ACLD. The NSQIP evaluates postoperative morbidities and 30-day mortality, but the prevalence of patients with cirrhosis is unknown.

Only two models have been developed to evaluate surgical risk in patients with ACLD: the Postoperative Mayo Risk Score (MRS) [11] and the Veterans Outcomes and Costs Associated with Liver Disease (VOCAL)-Penn Cirrhosis Surgical Risk Score [12,13]. The MRS included age, ASA score, MELD, and etiology of cirrhosis as independent predictors of surgical mortality during the short- (7, 30, and 90 days) and long-term (1 and 5 years). Nevertheless, it did not include the type of surgery [11]. The VOCAL-Penn is the most recent and complete surgical risk score to evaluate patients with cirrhosis, including variables such as age, liver function (bilirubin and albumin levels), portal hypertension (platelet count), etiology of cirrhosis (non-alcoholic fatty liver disease), comorbidities (obesity and ASA score), and the type and emergency of surgery [12,13]. Although the VOCAL-Penn improves mortality risk prediction over previous scores (CTP, MELD, MRS), it has not been validated in European cohorts.

Therefore, the primary aim of our study was to compare the surgical risk mortality prediction of the existing risk scores (CTP, MELD-Na, MRS, NSQIP, and VOCAL-Penn) in a European cohort of patients with ACLD. As secondary aims, we described comorbidity, complications after surgery, and variables related to mortality in our cohort.

## 2. Materials and Methods

### 2.1. Study Design and Population

This is a retrospective, observational, single-center study (Hospital del Mar) of patients with ACLD who underwent major surgery between January 2010 and December 2019. Patients were identified in the hospital registry by cross-matching the International Classification of Diseases (ICD) related to CLD, cirrhosis, its complications [ICD-9 571, 572.2, 572.3, 572.4, 572.5, 573.5, 070.2, 070.3, 070.44, 070.6, 070.7, and ICD-10 K70, K72.10/K72.11, K73, K74, K75.4, K75.9, K76.0, K76.1, K76.6, K76.7, K76.81, B19.0], and the Common Procedure Terminology (CPT) codes of all surgical procedures.

The hospital registry of patients with CLD was revised by a medical student (A.S.) who was supervised by the multidisciplinary team. The comorbidities were reviewed by a pharmacist (E.C.). Surgical procedures were scrutinized by an expert surgeon (A.P.) and anesthesiologist (J.A). Two expert hepatologists (L.C. and J.A.C) revised all the liver-related data and the ACLD categorization. Only patients with the inclusion criteria for ACLD who underwent major surgeries counted in the VOCAL-Penn [13] were included.

ACLD was defined by the presence of any chronic liver disease and at least one of the following criteria: (1) thrombocytopenia with platelets <150,000/uL and splenomegaly [14]; (2) an AST-to-Platelet Ratio Index (APRI) >2 [15]; (3) a nodular liver edge seen via ultrasound [16]; (4) transient elastography (TE) of >15 kPa [17]; and/or (5) signs of portal hypertension seen via upper digestive endoscopy (UDE) [18].

The major surgeries were classified according to (1) the localization, abdominal (laparoscopic or laparotomy), abdominal wall, vascular, orthopedic, and thoracic/cardiac, and (2) the emergency indication (urgent or elective). Emergent surgery was considered performed within the first 24 h after the diagnosis of the surgical pathology. Early reoperation was defined as a second surgery related to a complication of the initial surgery and performed on the same admission or in the first postoperative month.

We excluded patients with (1) ASA-V because of its intrinsic high mortality risk. (2) early reoperations, and (3) surgeries not included in the VOCAL-Penn (localized in the central nervous system, hepatic surgeries, or those with accepted low risk).

The study protocol was approved by the Ethical Committee of our institution, ‘Comitè Ètic d’Investigació Clínica -Parc de Salut Mar’, study reference 2020/9640, and by the ethical guidelines of the 1975 Declaration of Helsinki.

### 2.2. Data Collection, Mortality Estimation Risks, and Definitions

Sociodemographic data (age and gender), the dates of admission, surgery, and hospital discharge, and hospital stay were obtained through the hospital registry. Information about ACLD (etiology, decompensation, presence of ascites 30 days prior to surgery, TE, and UDE at an interval of 2 years) was retrospectively obtained from medical records. Laboratory data on liver function (bilirubin, albumin, prothrombin time, INR, and platelets) and renal function (urea, creatinine, and sodium) were collected at an interval of less than six months.

We calculated the CTP [5], MELD [19], and MELD-Na [20] scales based on the published formulas. Comorbidities were evaluated by ASA score [8] and CirCom [9]. The VOCAL-Penn was calculated based on the divulged formula [13]. The MRS [11] and NSQIP [10] mortality estimation risks were obtained according to online calculators: https://www.mayoclinic.org/medical-professionals/transplant-medicine/calculators/post-operative-mortality-risk-in-patients-with-cirrhosis/itt-20434721 and https://riskcalculator.facs.org/RiskCalculator/, (accessed on 20 January 2021) respectively. The postoperative mortality predicted by the surgical scores is available according to their design: the VOCAL-Penn score at 30, 90, and 180 days, the MRS at 30 and 90 days, and the NSQIP at 30 days.

After surgery, we evaluated renal function (maximum creatinine and creatinine at discharge), data regarding bacterial infection (type and severity), ACLD decompensation (ascites, encephalopathy, and portal hypertension bleeding), and hemorrhage (type and need for transfusion of blood products) during admission. The development of acute kidney injury (AKI) was characterized by the Kidney Disease Improving Global Guidelines [21]. We defined severe bacterial infection as requiring intensive care unit (ICU) admission for organ support (vasoactive drugs, mechanical ventilation, and renal replacement therapy). A multidrug-resistant microorganism (MDRM) was identified if it presented resistance to ≥2 groups of antibiotics. We also recorded worsened renal function, the presence of bacterial infection, ACLD decompensation, and/or hemorrhage from discharge to 90 days after surgery. The date and cause of death were compiled, and we calculated whether it occurred within 30 days, 90 days, or 180 days after surgery.

### 2.3. Statistical Analysis

Categorical variables were described as frequencies and percentages. Continuous variables were detailed as medians and interquartile ranges (IQR). We contrasted the baseline characteristics of our cohort (H. Mar) to those reported in the original study that created the VOCAL-Penn score [13] by comparing the proportions of categorical variables and medians of continuous variables with the Wilcoxon signed-rank test.

We assessed variables related to 90-day mortality in our cohort. Variables were compared between groups using χ^2^ for categorical variables and the Mann-Whitney U test for continuous variables. Covariates that were significant with *p* < 0.05 in univariate analysis were included in multivariate forward stepwise Cox regression models. A maximum of one variable for every 10 events was entered into the model. A Kaplan-Meier analysis and Log-Rank test were used to gauge the association between the variables of interest and the observed postoperative mortality.

Surgical risk scores (CTP, MELD-Na, MRS, NSQIP, and VOCAL-Penn) were evaluated using tools of discrimination and calibration. The discrimination or predictive capacity for mortality of each score was depicted graphically according to their median (IQR) mortality rate in patients with the presence or absence of observed mortality after surgery at 30 days (by MRS, NSQIP, and VOCAL-Penn), 90 days (by MRS and VOCAL-Penn), and 180 days (by VOCAL-Penn). The predictive capacity of the scores was estimated using Receiver Operating Characteristic (ROC) curves and concordance statistics (c-statistics) with 95% confidence intervals (CIs). The accuracy was considered excellent if the c-statistic was >0.9 and good for values between 0.7 and 0.9 [22]. Moreover, the c-statistic (95%CI) of each score was compared to VOCAL-Penn (as the reference) according to the Hanley and MacNeil test [23] to identify those with the highest diagnostic accuracy. Calibration or goodness of fit to the observed mortality was evaluated with a graph of the observed events rate against the predicted mortality probabilities of each score. The overall performance of the scores was studied via the Brier score, which is a global measure that incorporates discrimination and calibration information. Its value can range from 0 to 1. The lower the value, the better the prediction.

All the analyses were 2-tailed. Statistical analysis and graphs were performed using IBM SPSS statistics V 24.0 (SPSS Inc., Chicago, IL, USA) and STATA V15.1 (StataCorp LLC., College Station, TX, USA).

## 3. Results

### 3.1. Study Population and Comparison with the VOCAL-Penn Cohort

**Figure 1:**
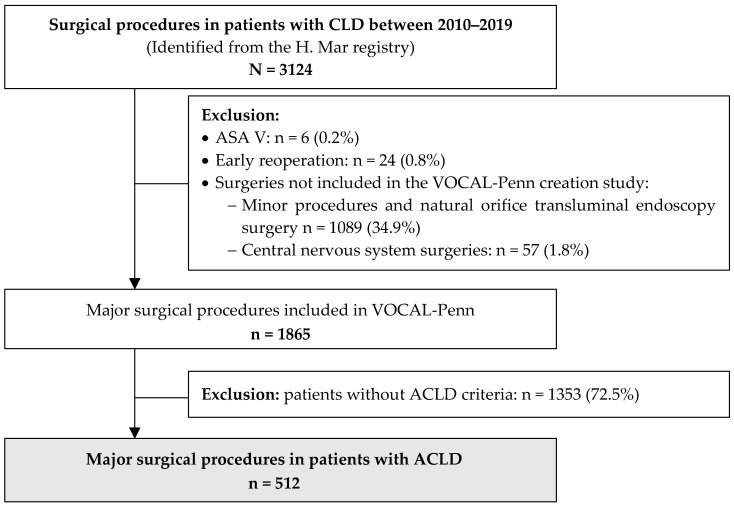
Flowchart of the included patients. CLD: Chronic Liver Disease; ASA: American Society of Anesthesiologists physical status classification system; ACLD: Advanced Chronic Liver Disease.

All surgical procedures in patients with CLD between January 2010 and December 2019 (N = 3124) were initially evaluated. Only the major surgical procedures included in VOCAL-Penn (n = 1865) were considered. Patients without ACLD (n = 1353) were excluded. Therefore, after a profound revision of the hospital registry, 512 patients with ACLD who underwent a major surgical procedure were included. The flowchart of the study population is shown in Figure 1.

Demographic data, information about ACLD, and the comorbidities of the included patients (n = 512) are summarized in Table 1. The median (IQR) age was 66 years (57–75), and 332 (64.8%) patients were male. The etiologies of ACLD were alcohol (45.3%), viral (31.4%), and metabolic-associated fatty liver disease (MAFLD) (10.8%). Endoscopic signs of portal hypertension were present in 58.5%. Most patients had good liver function before surgery: 70.7% were CTP A, and the median (IQR) MELD-Na was 12 (8–16), while 29.3% had a history of a previous decompensation (15.2% with ascites 30 days before surgery). Of the surgeries, 40.2% were urgent, and the predominant localizations were abdominal (42.6%), orthopedic (25.0%), and abdominal wall (21.7%). Urgent surgeries were performed more frequently in patients with CTP B/C (60.2%) than in CTP A (29.6%) (*p* < 0.001) and in those with ascites (70.5%) than in those without (34.8%) (*p* < 0.001).

Several disparities were detected when comparing the H. Mar and VOCAL-Penn cohorts (Table 1). The VOCAL-Penn cohort had a clear predominance of males (97.2%) compared to H. Mar (64.8%) (*p* < 0.001) and included patients with better liver function as evidenced by the MELD, MELD-Na, and CTP scales (*p* < 0.001 in all variables). However, the proportion of ascites before surgery was similar between both cohorts (H. Mar 15.2% vs. VOCAL-Penn 13.2%; *p* = 0.174). The predominant etiologies of ACLD in the H. Mar were alcohol and hepatitis C, while in the VOCAL-Penn cohort, they were alcohol and its combination with hepatitis C. The presence of metabolic and cardiovascular comorbidities, such as diabetes, obesity, and hypertension, was significantly higher in the VOCAL-Penn cohort, as well as the rate of previous decompensation (H. Mar 29.3% vs. VOCAL-Penn 43.8%; *p* < 0.001). Therefore, the prevalence of patients with ASA-IV was higher in the VOCAL-Penn cohort (54.4%) than in H. Mar (24.2%) (*p* < 0.001). Emergent and abdominal (laparoscopic and laparotomy) surgeries were more frequent in the H. Mar cohort, while abdominal wall, vascular, and thoracic/cardiac surgeries were predominant in VOCAL-Penn Cohort.

### 3.2. Clinical Events and Mortality after Surgery

During hospitalization, 38 (7.4%) patients died. Liver decompensation was observed in 149 (29.1%) patients, ascites in 141 (27.5%), encephalopathy in 40 (7.8%), and hemorrhage due to portal hypertension in 7 (1.4%). Bacterial infections were observed in 200 (39.1%) patients. The initial infection was spontaneous bacterial peritonitis (SBP) in 57 (28.5%), urinary infection in 42 (21.0%), other intra-abdominal infections in 35 (17.5%), and respiratory infection in 27 (13.5%). Seventy-nine (39.5%) were caused by MDRM, and 47 (23.5%) developed a severe bacterial infection: 19 (40.4%) SBP, 13 (27.7%) respiratory infections, 10 (21.3%) bacteremia, and 5 (10.6%) other intra-abdominal infections. Hemorrhage presented in 73 (14.3%) patients: non-digestive in 46 (63.0%), digestive non-portal hypertension in 20 (27.4%), and due to portal hypertension in 7 (9.6%). The transfusion of at least two red blood cell packs was required in 56 (76.7%) patients with hemorrhage. Renal function was evaluated in 427 patients, and 124 (29.0%) developed AKI during admission: 25 (20.2%) grade 1A, 43 (34.7%) grade 1B, 25 (20.2%) grade II, and 31 (25.0%) grade III. Ascites was more frequent in patients with AKI (55.6%) compared to those without (22.4%) (*p* < 0.001).

After discharge and during the first 90 days of follow-up after surgery, 12 (2.3%) patients died, and 135 (26.4%) had ascites: clinical in 68 (14.4%) and radiological in 67 (14.2%). Seventy-eight (16.6%) patients showed some bacterial infection: urinary in 24 (30.8%), respiratory in 15 (19.2%), and cellulitis or skin in 15 (19.2%). Thirty-two (6.8%) patients presented with bleeding, mainly of soft tissue. Renal function was evaluated in 349 patients, and AKI was observed in 34 (9.7%). Therefore, 158 (45.2%) patients presented with AKI during the first 90 days after surgery.

The mortality rate at 30, 90, and 180 days from surgery was 6.4% (n = 33), 9.8% (n = 50), and 13.7% (n = 70), respectively. Liver decompensation, sepsis, and cardiovascular events were the most frequent causes of death at the three time points. Mortality rates by liver decompensation, sepsis, and cardiovascular events at 90 days were 18 (36.0%), 11 (22.0%), and 8 (16.0%), respectively. However, as we moved away from the date of surgery, patients died less from liver decompensation and more from cancer or unknown causes.

### 3.3. Variables Related to Mortality after Surgery

**Figure 2:**
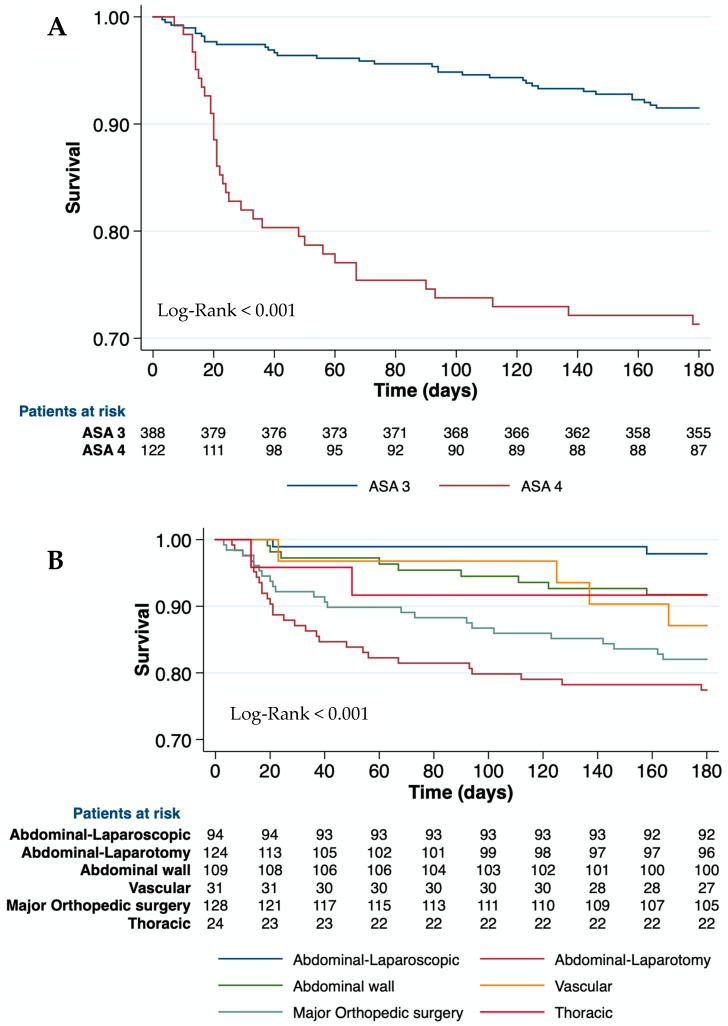

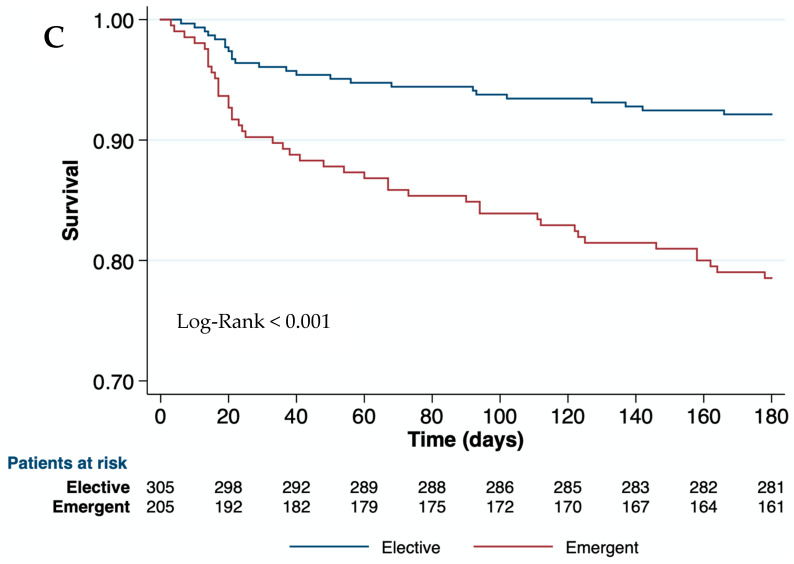
Postoperative mortality (Kaplan-Meier analysis) according to ASA (**A**), type (**B**), and emergency (**C**) of surgery. ASA: American Society of Anesthesiologists physical status classification system.

A univariate analysis of the variables related to 90-day mortality is depicted in Table 2. In our cohort, the etiology of the ACLD did not impact mortality. Neither obesity nor other metabolic comorbidities were useful in discriminating the risk of mortality. Age and liver-related variables, such as bilirubin, INR, albumin, platelets, and ascites, 30 days before surgery were associated with 30-day, 90-day, and 180-day mortality. The baseline creatinine level was related to 30- and 90-day mortality but not to 180-day mortality. Chronic kidney disease was associated with only 30-day mortality and not with 90- or 180-day mortality. Moreover, the CirCom score was not useful for predicting mortality (Log-Rank_30D_ = 0.133; Log-Rank_90D_ = 0.531; and Log-Rank_180D_ = 0.566). However, patients with ASA-IV showed higher postoperative mortality than those with ASA-III at all-time points (Table 2 and Figure 2A). The type and the emergency of surgery were associated with postoperative mortality (Log-Rank< 0.01 at all-time points). Therefore, open abdominal surgeries and major orthopedic surgeries had the highest mortality risk at 90 days (46.0% and 30.0%, respectively) (Table 2 and Figure 2B,C). Clinical events during hospitalization, such as AKI, bacterial infections, and hemorrhage, were also related to mortality at all time points (*p* < 0.001) (Table 2).

Variables associated with 90-day postoperative mortality in the multivariate Cox regression analysis are depicted in Table 3. The variables were grouped into four categories: (1) age and comorbidity, (2) liver function, (3) type and urgency of surgery, and (4) clinical events during admission. In the first group, age and ASA-IV were related to mortality at all three time points. Neither chronic kidney disease nor baseline creatinine were independently associated with mortality. Regarding liver function, bilirubin and albumin were associated with mortality at all time points and INR at 90 days. No differences in the model were found when choosing ascites or platelet levels. In the third category, patients undergoing urgent surgery showed between 2–3 times higher risk of mortality, and those undergoing open abdominal surgery or major orthopedic surgery developed a higher mortality risk at all three time points. Finally, patients with AKI or bacterial infections during admission showed a higher risk of mortality during the first 90 days after surgery.

### 3.4. Diagnostic Accuracy and Calibration of Surgical Risk Scales

**Figure 3:**
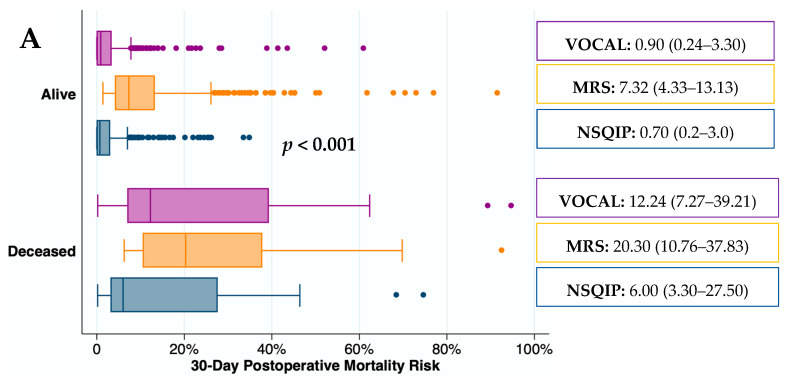

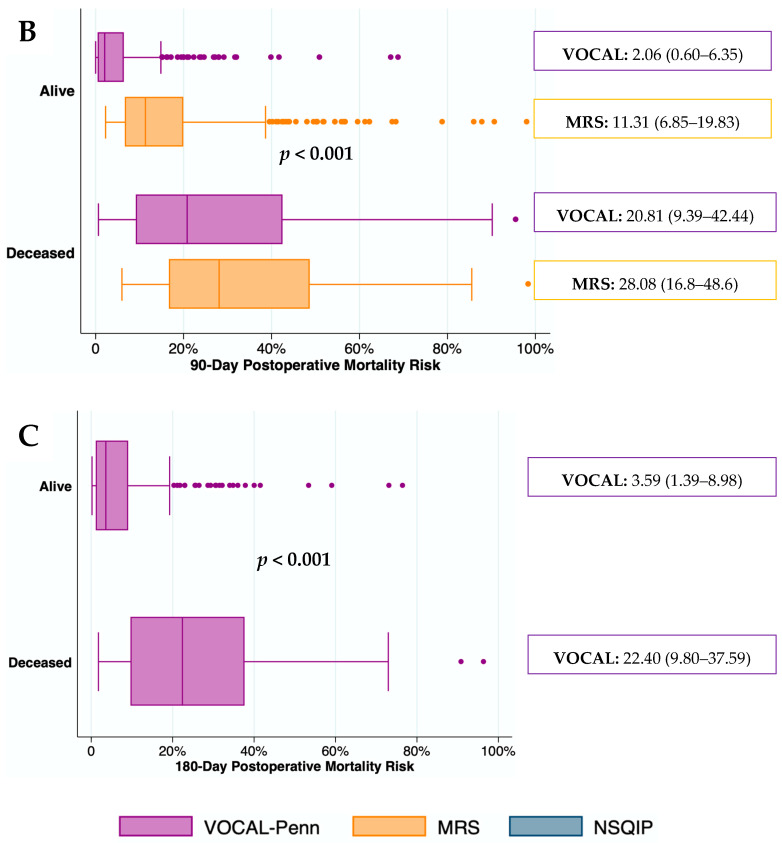
Predicted mortality by VOCAL-Penn, MRS, and NSQIP scores at 30 (**A**), 90 (**B**), and 180 (**C**) days according to observed mortality.

The predictive capacity for mortality of the surgical risk scores is depicted graphically in Figure 3. The median (IQR) mortality rate was significantly higher in deceased patients than in those who remained alive at all time points (*p* < 0.001). The median (IQR) mortality risk predicted by the MRS in patients who remained alive 30 and 90 days after surgery was higher than predicted by the VOCAL-Penn. In contrast, the median (IQR) mortality risk predicted by the NSQIP in deceased patients 30 days after surgery was lower than predicted by the VOCAL-Penn.

**Figure 4:**
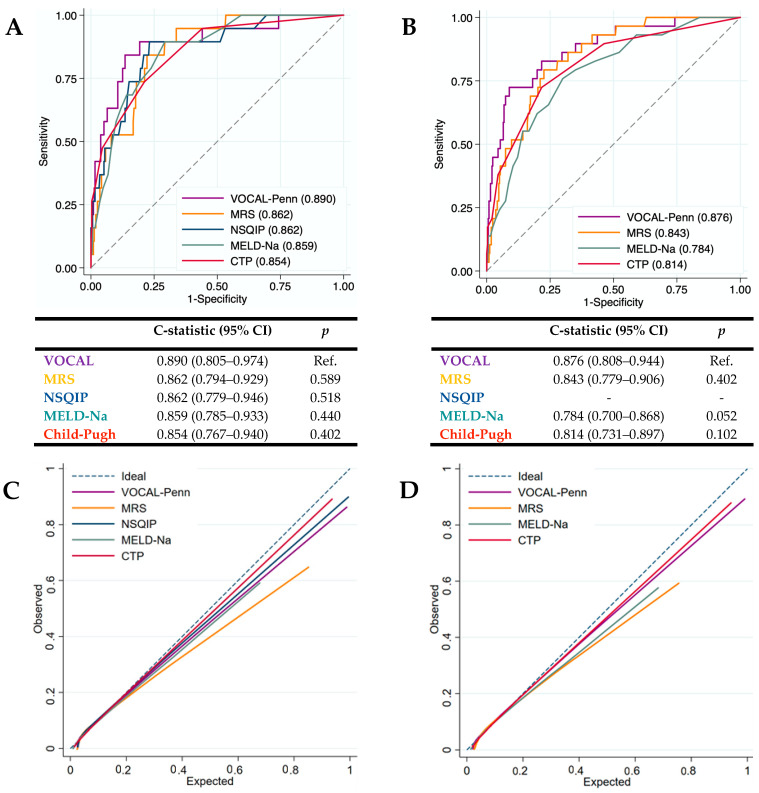
Diagnostic accuracy (ROC curves) of the surgical risk scales (Child-Pugh, MELD-Na, NSQIP, MRS, and VOCAL-Penn) for 30-day (**A**) and 90-day (**B**) postoperative mortality, and calibration curves for 30-day (**C**) and 90-day (**D**) postoperative mortality.

The diagnostic accuracy of the surgical risk scales (CTP, MELD-Na, NSQIP, MRS, and VOCAL-Penn) for identifying 30- and 90-day postoperative mortality was evaluated in our cohort (Figure 4A,B). VOCAL-Penn showed a good predictive capacity for mortality at 30 days (C-statistic_VP-30D_ = 0.890) and 90 days (C-statistic_VP-90D_ = 0.876). The c-statistic (95%CI) of each score was compared with VOCAL-Penn (as the reference). Even though VOCAL-Penn presented a higher c-statistic than the rest of the scores, the differences with MRS, NSQIP, MELD-Na, and CTP were not statistically significant.

Calibration curves (Figure 4C,D) showed that CTP and VOCAL-Penn have better calibration than MRS at 30 and 90 days because MRS overestimates postoperative mortality. MELD-Na has a better calibration at 30 days than at 90 days.

The overall performance of the surgical risk scales was evaluated via the Brier score (Table 4). The Brier score at 30 and 90 days after surgery for VOCAL-Penn (Brier_VP-30D_ = 0.046 and Brier_VP-90D_ = 0.055) was similar to NSQIP (Brier_NSQIP-30D_ = 0.044) and lower than MRS (Brier_MRS-30D_ = 0.058 and Brier_MRS-90D_ = 0.081), MELD-Na (Brier_MELD-30D_ = 0.058 and Brier_MELD-90D_ = 0.082), and Child-Pugh (Brier_CTP-30D_ = 0.050 and Brie_CTP-90D_ = 0.074).

### 3.5. Diagnostic Accuracy of VOCAL-Penn in Different Scenarios

**Figure 5:**
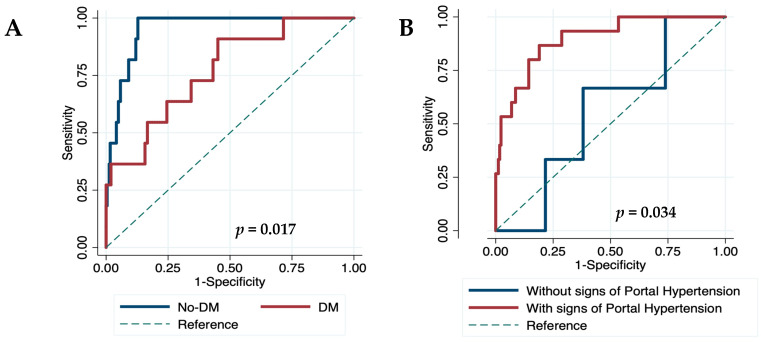
Diagnostic accuracy (ROC curves) of VOCAL-Penn at 30 days according to diabetes mellitus (**A**) and portal hypertension signs on upper digestive endoscopy (**B**). DM: diabetes mellitus.

Finally, we evaluated the diagnostic accuracy of VOCAL-Penn for identifying 30-, 90-, and 180-day postoperative mortality in different scenarios. The diagnostic accuracy of VOCAL-Penn did not reveal differences according to gender, etiology of ACLD, or the presence of chronic kidney disease. However, VOCAL-Penn showed a lower discrimination capacity at 30 days in patients with diabetes (C-statistic_30D_ = 0.770) compared to those without (C-statistic_30D_ = 0.953) (*p* = 0.017) (Figure 5A), in patients without endoscopic signs of portal hypertension (C-statistic_30D_ = 0.555) compared to those with (C-statistic_30D_ = 0.898) (*p* = 0.034) (Figure 5B), and for abdominal wall surgeries (C-statistic_30D_ = 0.608) compared to abdominal (C-statistic_30D_ = 0.916) or orthopedic (C-statistic_30D_ = 0.948) surgeries (*p* < 0.05 in both cases). A decrease in diagnostic accuracy according to the category of surgery was also found 90 and 180 days after surgery. Similarly, its discrimination capacity at 180 days was lower for urgent (C-statistic_180D_ = 0.692) compared to elective (C-statistic_180D_ = 0.901) surgeries (*p* = 0.008).

## 4. Discussion

The postoperative mortality of patients with cirrhosis is an important area of improvement for professionals who participate in the care of these patients, especially considering that the need for surgical interventions has increased with age and comorbidity. This has motivated recent clinical guidelines on perioperative management of patients with ACLD [24,25,26].

After more than a decade without new tools for evaluating surgical risk in patients with cirrhosis, in 2021, the VOCAL-Penn score was designed based on a large American cohort [13]. Our European cohort of patients with ACLD has shown a mortality rate after surgery similar to the American cohorts [12,13]. Liver decompensation, sepsis, and cardiovascular events were the most frequent causes of death. However, substantial differences were found when comparing the H. Mar and VOCAL-Penn cohorts. Our European cohort showed (1) a more equilibrated distribution of males (64.8% vs. 97.2%), (2) alcohol consumption as the predominant etiology of ACLD, (3) a lower presence of metabolic and cardiovascular comorbidities, such as diabetes, obesity, and hypertension, with a lower representation of patients with ASA-IV (24.2% vs. 54.4%), and (4) a higher frequency of abdominal (laparoscopic and laparotomy) and urgent surgeries.

Studies that evaluate the postoperative mortality risk in patients with cirrhosis are mostly retrospective and heterogeneous regarding extrahepatic comorbidities and the invasiveness or type of surgery [25]. The mortality rate after surgery in patients with ACLD in our cohort was 6.4% at 30 days, 9.8% at 90 days, and 13.7% at 180 days, similar to those previously described in the American cohorts for the creation and validation of VOCAL-Penn [12,13]. We have data, especially for patients undergoing abdominal hernia surgery [27]. In 2019, Mahmud et al. published a retrospective study including more than 72,000 surgical procedures that showed a greater postoperative risk in major abdominal and cardiovascular surgeries [28]. From their work emerged the categorization of the type of surgery proposed for the VOCAL-Penn score. Furthermore, the study observed higher in-hospital mortality for all emergency surgeries except for cholecystectomy. Similarly to the studies evaluating the MRS [11] and VOCAL-Penn [12,13] scores, our study found that age, ASA scale, bilirubin, albumin, INR, open abdominal surgery, and urgent surgeries were variables independently associated with mortality in European patients. Additionally, unlike the original VOCAL-Penn study, major orthopedic surgery was also associated with mortality. Therefore, variables associated with mortality in our cohort could be grouped into four categories: (1) age and comorbidity (ASA scale), (2) liver function (bilirubin, albumin, and INR), (3) type and urgency of surgery, and (4) complications during admission (AKI and bacterial infections).

The CirCom score was not useful in assessing postoperative mortality in our study, probably because it was designed to assess the risk of long-term mortality associated with comorbidities. Classic liver function scores, such as CTP and MELD-Na, showed lower diagnostic accuracy than other scores in our cohort, similar to those previously published [13].

The discriminative ability of MRS was similar to VOCAL-Penn’s, but the median (IQR) mortality risk predicted by MRS in patients who remained alive 30 and 90 days after surgery was higher than predicted by VOCAL-Penn, showing an overestimation of the risk and high variability. Moreover, the Brier score showed higher values for MRS than for VOCAL-Penn, demonstrating a worsened calibration and capacity for predicting postoperative mortality. These findings could be explained, at least in part, by differences in the prevalence of comorbidities, CLD stage, and surgical invasiveness reported in 2007 [11].

For the first time, the NSQIP calculator [10] was evaluated exclusively in patients with ACLD. We found that NSQIP had good discrimination and calibration to predict mortality at 30 days, probably due to the significant prevalence of ascites before surgery in our cohort. However, the median (IQR) mortality risk at 30 days after surgery predicted by NSQIP in deceased patients was lower than predicted by VOCAL-Penn, showing an infra-estimation of the risk and a lowered accurate prediction. Therefore, we recommend evaluating NSQIP in multicenter, large cohorts of patients with ACLD before concluding its usefulness in this specific population.

The necessity for useful tools in patients with ACLD led to the design and validation of the VOCAL-Penn score in 2021 [12,13]. This American cohort included almost exclusively men with a high prevalence of metabolic comorbidities and ASA-IV. In contrast, our cohort included a higher proportion of patients with alcohol consumption and hepatitis C infection, undergoing more frequent abdominal or urgent surgeries. These baseline differences between the H. Mar and VOCAL-Penn cohorts could explain the null association of MAFLD and obesity with mortality in our cohort. Neither chronic kidney disease nor baseline creatinine was independently associated with mortality in the H. Mar cohort, and no differences in the model were found when choosing platelet levels. These variables are included in the VOCAL-Penn score but could be redundant in European cohorts with a lower presence of metabolic and cardiovascular comorbidities (obesity, diabetes, and hypertension). Despite baseline differences between the H. Mar and VOCAL-Penn cohorts, VOCAL-Penn showed a very good discrimination ability for predicting mortality at 30 and 90 days. The calibration curve for VOCAL-Penn was not excellent, but the Brier score was the lowest for predicting mortality at 90 days in our European cohort. Importantly, the VOCAL-Penn’s diagnostic accuracy was significantly lower in patients with diabetes (C-statistic_30D_ = 0.770), without signs of portal hypertension (C-statistic_30D_ = 0.555), who underwent abdominal wall surgery (C-statistic_30D_ = 0.608), or with urgent surgeries (C-statistic_180D_ = 0.692). However, a more detailed analysis of multicenter European cohorts is required to draw solid conclusions and establish preventive strategies.

Our study has some limitations. First, it was performed in a single center. However, our results are based on a large cohort of patients very well characterized by surgeons, anesthesiologists, pharmacists, and hepatologists. Second, it is a retrospective study that only evaluated patients who underwent surgery. Therefore, those who did not undergo surgery due to the perception of the multidisciplinary team that the available scales showed an unacceptably high risk were not included. Third, the lack of some variables led to the unavailability of some scores for some surgeries (vascular, thoracic, and cardiac) that could be underrepresented. However, the predictive capacity of the scales has been compared in the patients with all the data. In contrast, our study has important strengths and findings: (1) it is the first European cohort evaluating the VOCAL-Penn and NSQIP scales in a large, well-characterized cohort of patients with ACLD; (2) we found substantial differences when comparing European and American cohorts; (3) some variables included in VOCAL-Penn were not associated with mortality; (4) calibration of the VOCAL-Penn score was not excellent in our cohort; and (5) we identified patients (with diabetes or without signs of portal hypertension) and surgeries (abdominal wall and urgent) discriminated as suboptimal by the VOCAL-Penn score.

## 5. Conclusions

Our European cohort of patients with ACLD has shown a mortality rate after surgery similar to those previously described in American studies. Liver decompensation, sepsis, and cardiovascular events were the most frequent causes of death. However, substantial differences were found when compared to American cohorts. Some variables included in the VOCAL-Penn score were not associated with an increased risk of mortality. Consequently, the calibration of the VOCAL-Penn score was not excellent, and the discriminative ability decreased in some subgroups of our patients. We consider that our results should be validated in larger, multicenter, and extensive prospective studies to confirm these findings and construct new and more accurate surgical scores for European patients.

## Figures and Tables

**Table 1:** Comparison of H. Mar and VOCAL-Penn cohorts.

	H. Mar Cohort (n = 512)	VOCAL-Penn Cohort (n = 4712)	*p*
Age (years)	66 (57–75)	64 (60–69)	<0.001
Male sex, n (%)	332 (64.8)	4582 (97.2%)	<0.001
Etiology of liver disease, n (%) (n = 510)			
Hepatitis C	149 (29.2)	612 (13.0)	<0.001
Hepatitis B	11 (2.2)	73 (1.5)	0.227
Alcohol	231 (45.3)	1662 (35.3)	<0.001
MAFLD	52 (10.8)	585 (12.4)	0.255
Hepatitis C + Alcohol	36 (7.1)	1388 (29.5)	<0.001
Other	28 (5.5)	392 (8.3)	0.020
Creatinine (mg/dL)	0.87 (0.69–1.16)	1.0 (0.8–1.2)	<0.001
Total bilirubin (mg/dL) (n = 454)	0.82 (0.50–1.39)	0.8 (0.5–1.1)	<0.001
Albumin (g/dL) (n = 447)	4.0 (3.3–4.4)	3.7 (3.2–4.1)	0.005
INR (n = 509)	1.17 (1.08–1.32)	1.1 (1.0–1.2)	<0.001
Platelet count (·10^3^/μL) (n = 507)	132.0 (93.0–195.0)	152.0 (107.0–207.0)	0.013
MELD (n = 454)	10 (8–13)	8 (7–11)	<0.001
MELD-Na (n = 454)	12 (8–16)	10 (8–14)	<0.001
Child-Pugh class, n (%) (n = 420)			
A	297 (70.7)	4159 (88.3)	<0.001
B	104 (24.8)	530 (11.2)	<0.001
C	19 (4.5)	23 (0.5)	<0.001
Ascites 30 days before surgery, n (%)	78 (15.2)	620 (13.2)	0.174
History of previous decompensation, n (%)	150 (29.3)	2066 (43.8)	<0.001
Endoscopic signs of portal hypertension, n (%) (n = 422)	247 (58.5)	-	-
Hypertension, n (%)	242 (47.3)	3904 (85.4)	<0.001
Diabetes, n (%)	151 (29.5)	2355 (51.7)	<0.001
Obesity (BMI ≥ 30 kg/m^2^), n (%) (n = 454)	142 (31.3)	3359 (73.8)	<0.001
Chronic kidney disease, n (%)	77 (15.0)	-	-
Peripheral vascular disease, n (%)	41 (8.0)	-	-
Previous acute myocardial infarction, n (%)	26 (5.1)	1622 (34.4)	<0.001
Congestive heart failure, n (%)	29 (5.7)	1239 (26.3)	<0.001
Active cancer, n (%)	107 (20.9)	-	-
ASA-IV	124 (24.2)	2565 (54.4)	<0.001
Surgery category, n (%)			
Abdominal–Laparoscopic	94 (18.4)	47 (10.1)	<0.001
Abdominal–Open	124 (24.2)	665 (14.1)	<0.001
Abdominal wall	111 (21.7)	1308 (27.8)	0.002
Vascular	31 (6.1)	550 (11.7)	<0.001
Major orthopedic	128 (25.0)	1298 (27.5)	0.205
Thoracic/Cardiac	24 (4.7)	415 (8.8)	0.003
Emergent surgery, n (%)	206 (40.2)	476 (10.1)	<0.001

MAFLD: Metabolic Associated Fatty Liver Disease; INR: International Normalized Ratio; MELD: Model for End-Stage Liver Disease; BMI: Body Mass Index; ASA: American Society of Anesthesiologists physical status classification system.

**Table 2:** Univariate analysis of variables related to 90-day mortality after surgery.

	Live Patients (n = 462)	Deceased Patients (n = 50)	*p*
Age (years)	66 (56–75)	71 (63–81)	0.002
Male sex, n (%)	302 (65.4)	30 (60.0)	0.450
Etiology of liver disease, n (%) (n = 510)			
Viral	150 (32.5)	19 (38.8)	0.628
Alcohol	234 (50.8)	24 (49.0)	
MAFLD	50 (10.8)	5 (10.2)	
Other	27 (5.9)	1 (2.0)	
Creatinine (mg/dL)	0.86 (0.68–1.11)	1.11 (0.77–1.52)	0.002
Total bilirubin (mg/dL) (n = 454)	0.78 (0.50–1.31)	1.59 (0.90–3.22)	<0.001
Albumin (g/dL) (n = 447)	4.1 (3.4–4.4)	3.1 (2.5–3.7)	<0.001
INR (n = 509)	1.16 (1.07–1.29)	1.34 (1.18–1.72)	<0.001
Platelet count (·10^3^/μL) (n = 507)	135.0 (96.0–204.5)	101.0 (72.0–146.3)	0.001
MELD (n = 454)	9 (8–12)	14 (11–20)	<0.001
MELD-Na (n = 454)	11 (8–15)	18 (14–22)	<0.001
CTP class, n (%) (n = 420)			
A	285 (75.0)	12 (30.0)	<0.001
B	84 (22.1)	20 (50.0)	
C	11 (2.9)	8 (20.0)	
Ascites 30 days before surgery, n (%)	58 (12.6)	20 (40.0)	<0.001
History of previous decompensation, n (%)	123 (26.6)	27 (54.0)	<0.001
Endoscopic signs of portal hypertension, n (%) (n = 422)	222 (57.4)	25 (71.4)	0.033
Hypertension, n (%)	216 (46.8)	26 (52.0)	0.480
Diabetes, n (%)	135 (29.2)	16 (32.0)	0.682
Obesity (BMI ≥ 30 kg/m^2^), n (%) (n = 454)	133 (31.9)	9 (24.3)	0.341
Chronic kidney disease, n (%)	66 (14.3)	11 (22.0)	0.147
Peripheral vascular disease, n (%)	36 (7.8)	5 (10.0)	0.585
History of acute myocardial infarction, n (%)	23 (5.0)	3 (6.0)	0.755
Congestive heart failure, n (%)	23 (5.0)	6 (12.0)	0.041
Active cancer, n (%)	96 (20.8)	11 (22.0)	0.840
ASA-IV, n (%)	91 (19.7)	33 (66.0)	<0.001
CirCom, n (%)			
0	200 (43.3)	20 (40.0)	0.427
1 + 0 or 1 + 1	108 (23.4)	8 (16.0)	
3 + 0 or 3 + 1	136 (29.4)	19 (38.0)	
5 + 0 or 5 + 1	18 (3.9)	3 (6.0)	
Surgery category, n (%)			
Abdominal–Laparoscopic	93 (20.1)	1 (2.0)	0.001
Abdominal–Open	101 (21.9)	23 (46.0)	
Abdominal wall	103 (22.3)	8 (16.0)	
Vascular	30 (6.5)	1 (2.0)	
Major orthopedic	113 (24.5)	15 (30.0)	
Thoracic	22 (4.8)	2 (4.0)	
Emergent surgery, n (%)	174 (37.7)	32 (64.0)	0.001
Hepatic decompensation, n (%)	115 (24.7)	34 (68.0)	<0.001
Acute kidney injury during admission, n (%) (n = 427)	89 (23.5)	35 (71.4)	<0.001
AKI 1A	24 (6.3)	1 (2.0)	<0.001
AKI 1B	35 (9.3)	8 (16.3)	
AKI 2	17 (4.5)	8 (16.3)	
AKI 3	13 (3.4)	18 (36.7)	
Infection during admission, n (%)	158 (34.2)	42 (84.0)	<0.001
Severe infection during admission, n (%) (n = 200)	26 (16.5)	21 (50.0)	<0.001
Hemorrhage during admission, n (%)	57 (12.3)	16 (32.0)	<0.001
Hemorrhage with transfusion of ≥RBC packs, n (%)	41 (8.9)	15 (30.0)	<0.001

MAFLD: Metabolic Associated Fatty Liver Disease; INR: International Normalized Ratio; MELD: Model for End-Stage Liver Disease; BMI: Body Mass Index; ASA: American Society of Anesthesiologists physical status classification system; CirCom: Cirrhosis Comorbidity score; AKI: Acute Kidney Injury; RBC: red blood cell.

**Table 3:** Multivariate Cox regression analysis to evaluate variables independently associated with the 90-day postoperative mortality.

Variable	HR	95% CI	*p*
Age	1.034	1.009–1.058	0.006
Congestive heart failure			0.677
ASA-IV	6.829	3.801–12.267	<0.001
Bilirubin	1.072	1.008–1.140	0.028
Albumin	0.336	0.229–0.494	<0.001
INR	2.275	1.079–4.797	0.031
Platelets			0.690
Surgery category			
Abdominal-Laparoscopic	Ref.		
Abdominal-Open	19.437	2.629–143.700	0.004
Abdominal wall	5.937	0.730–48.255	0.096
Vascular	3.926	0.245–63.017	0.334
Major orthopedic	12.685	1.675–96.081	0.014
Thoracic	14.511	1.270–165.758	0.031
Emergent surgery	2.801	1.530–5.126	0.001
Hepatic decompensation			0.061
AKI	4.592	2.418–8.721	<0.001
Infection	4.936	2.162–11.270	<0.001
Hemorrhage			0.171

ASA: American Society of Anesthesiologists physical status classification system; INR: International Normalized Ratio; AKI: Acute Kidney Injury.

**Table 4:** C-statistic and Brier scores for VOCAL-Penn, MRS, and NSQIP at 30 and 90 days.

	C-Statistic (95% CI)		Brier Score	
	30-Day Postoperative Mortality	90-Day Postoperative Mortality	30-Day Postoperative Mortality	90-Day Postoperative Mortality
VOCAL-Penn	0.890 (0.805–0.974)	0.876 (0.808–0.944)	0.046	0.055
MRS	0.862 (0.794–0.929)	0.843 (0.779–0.906)	0.058	0.081
NSQIP	0.862 (0.779–0.946)	-	0.044	-
MELD-Na	0.859 (0.785–0.933)	0.784 (0.700–0.868)	0.058	0.082
Child-Pugh	0.854 (0.767–0.940)	0.814 (0.731–0.897)	0.050	0.074

VOCAL: Veterans Outcomes and Costs Associated with Liver Disease; MRS: Postoperative Mayo Risk score (MRS); NSQIP: National Surgery Quality Improvement Program; MELD: Model for End-Stage Liver Disease.

## Data Availability

Data is unavailable due to ethical restrictions.
